# Supplementary material for: Association of Coffee and Energy Drink Intake with Suicide Attempts and Suicide Ideation: A Systematic Review and Meta-Analysis
Source: Nutrients. 2025 Jun 2;17(11):1911. doi: 10.3390/nu17111911 (PMC12157705; doi:10.3390/nu17111911)
Supplement: Supplementary file 1 [file nutrients-17-01911-s001.zip › nutrients-3660723-supplementary.pdf]

Association of coffee and energy drink intake with suicide attempts and suicide ideation: A systematic review and meta-analysis

Supplementary Table S1: Search Strategy
Supplementary Table S2: Mixed effects meta-regression of pooled OR against amount of energy drink consumption per month and association with suicidality outcomes
Supplementary Table S3: Mixed effects meta-regression of pooled RR against amount of coffee consumption per month and association with suicide attempts
Supplementary Table S4: Evaluation of the mediating or confounding effect of other substance abuse on caffeine intake and suicidality outcomes
Supplementary Table S5: Evaluation of the mediating or confounding effect of gender on caffeine intake and suicidality outcomes
Supplementary Table S6: Quality assessment of included cohort studies using the Joanna Brigg's Institute Critical Appraisal tool
Supplementary Table S7: Certainty of evidence assessed using the GRADE framework

Supplementary Table S1: Search Strategy

PubMed

#1	"Caffeine"[Mesh] OR "Coffee"[Mesh] OR "Tea"[Mesh] OR "Energy Drinks"[Mesh] OR "1,3,7-Trimethylxanthine"[tiab] OR “Caffedrine”[tiab] OR "No Doz"[tiab] OR "Coffea"[tiab] OR "Green Tea"[tiab] OR "Black Tea"[tiab]
#2	("Self-Injurious Behavior"[Mesh] OR "Suicide"[Mesh] OR "Suicide, Attempted"[Mesh] OR "Suicide, Completed"[Mesh] OR "Suicidal Ideation"[Mesh] OR Suicid* [tiab] OR “Suicidal Ideation” [tiab] OR “Self-harm” [tiab] OR “Self-injur*” [tiab] OR SIB [tiab] OR “deliberate self-harm” [tiab] OR DSH [tiab] OR parasuicide [tiab] OR “self-wounding” [tiab] OR “self-mutilation” [tiab] OR “auto-aggression” [tiab])

#1 and #2

Embase

#1	'caffeine'/exp OR 'coffee'/exp OR 'tea'/exp OR 'energy drink'/exp OR ‘green tea’:ti,ab OR ‘black tea’:ti,ab OR ‘tea extract’:ti,ab OR ‘tea infusion’:ti,ab OR ‘animine’:ti,ab OR ‘guaranine’:ti,ab OR ‘No Doz’:ti,ab
#2	(Suicid*:ti,ab OR 'Suicidal Ideation':ti,ab OR 'Self-harm':ti,ab OR 'Self-injur*':ti,ab OR SIB:ti,ab OR 'deliberate self-harm':ti,ab OR DSH:ti,ab OR parasuicide:ti,ab OR 'self-wounding':ti,ab OR 'self-mutilation':ti,ab OR 'auto-aggression':ti,ab OR 'automutilation'/exp OR 'suicide'/exp OR 'suicidal behavior'/exp OR 'suicide attempt'/exp OR 'suicidal ideation'/exp)

#1 and #2

Supplementary Table S2: Mixed effects meta-regression of pooled OR against amount of energy drink consumption per month and association with suicidality outcomes

	Estimate	p-value	95% CI	I^2 (% residual heterogeneity)
Suicide Attempts	0.0352	<0.0001	0.0233; 0.0470	61
Suicide Ideation	0.0376	0.0440	0.0010; 0.0743	99

Supplementary Table S3: Mixed effects meta-regression of pooled RR against amount of coffee consumption per month and association with suicide attempts

	Estimate	p-value	95% CI	I^2 (% residual heterogeneity)
Suicide Attempts	-0.0085	0.0249	-0.0160; -0.0011	63

Supplementary Table S4: Evaluation of the mediating or confounding effect of other substance abuse on caffeine intake and suicidality outcomes

Author	Year	Country	Study population	Key findings†
Baethge	2009	Germany	352 sardinian bipolar disorder patients with a mean age of 44.5 ± 14.7 years followed up at the Lucio Bini Mood Disorders Center were evaluated using a Structured Clinical Interview from February 2002 to December 2007.	Compared to non-coffee drinkers, coffee drinkers were more likely to have alcohol use (OR = 1.73, 95%CI = 1.02, 2.93).<br><br>Compared to non-coffee drinkers, coffee drinkers were more likely to have cigarette use (OR = 1.04, 95%CI = 1.02, 1.07).
Evren	2015	Istanbul	4957 10th grade students with a mean age of 15.56 years old (± 2.74 years old), across 45 schools from 15 districts in Istanbul, were evaluated through a cross-sectional online self-report survey.	Compared to those who do not consume energy drinks, those who consumed energy drinks once in their lifetime were more likely to have lifetime tobacco use (OR = 2.62, 95%CI = 2.28, 3.02), more likely to have lifetime alcohol use (OR = 2.78, 95%CI = 2.37, 3.25) and more likely to have lifetime drug use (OR = 2.09, 95%CI = 1.59, 2.75).<br><br>Compared to those who do not consume energy drinks, those who consumed energy drinks once to three times in a month were more likely to have lifetime tobacco use (OR = 5.62, 95%CI = 4.80, 6.57), more likely to have lifetime alcohol use (OR = 4.79, 95%CI = 4.08, 5.64) and more likely to have lifetime drug use (OR = 3.57, 95%CI = 2.74, 4.65).<br><br>Compared to those who do not consume energy drinks, those who consumed energy drinks once to five times in a week were more likely to have lifetime tobacco use (OR = 10.53, 95%CI = 7.28, 15.22), more likely to have lifetime alcohol use (OR = 9.49, 95%CI = 6.94, 12.98) and more likely to have lifetime drug use (OR = 11.62, 95%CI = 8.16, 16.55).<br><br>Compared to those who do not consume energy drinks, those who consumed energy drinks everyday were more likely to have lifetime tobacco use (OR = 10.25, 95%CI = 5.91, 17.78), more likely to have lifetime alcohol use (OR = 9.88, 95%CI = 6.21, 15.71) and

				more likely to have lifetime drug use (OR = 24.22, 95%CI = 15.13, 38.76).
S. Park	2016	South Korea	68,084 adolescents aged 12-18, with a mean age of 15.09 years, were evaluated in a cross-sectional study in 2015, with data taken from the 2015 Korean Youth Risk Behaviour Web-Based Survey.	Compared to those who had energy drinks less than once a week, those who had energy drinks 5 times or more per week were more likely to drink alcohol (OR = 1.35, 95%CI = 1.40, 1.45).
Kwak	2021	South Korea	267,907 middle and high school students with a mean age of 15.0 at the time of assessment were evaluated in a cross-sectional study, with data taken from the Korean Youth Risk Behaviour Web-Based Survey from 2014-2017.	Participants who used tobacco were more likely to consume highly caffeinated beverages (HCB) (OR = 1.11, 95%CI = 1.06, 1.16).<br><br>Participants who consumed alcohol were more likely to consume highly caffeinated beverages (HCB) (OR = 1.17, 95%CI = 1.13, 1.22).
Akanni	2017	Nigeria	465 randomly selected secondary school students aged 16-19, with a mean age of 16.9, were evaluated in a cross-sectional study.	Students who consumed alcohol were significantly more likely to consume caffeine (OR = 3.41, 95%CI = 2.31, 5.02)<br><br>Students who used marijuana were significantly more likely to consume caffeine (OR = 5.94, 95%CI = 1.29, 27.43)<br><br>Students who smoked cigarettes were significantly more likely to consume caffeine (OR = 3.28, 95%CI = 1.64, 6.56)

Abbreviations: aOR, adjusted odds ratio; OR, odds ratio; CI, confidence interval; HR, hazard ratio

†Outcomes of interest include logistic or linear regression analysis for any association between other substance abuse on caffeine intake and suicidality outcomes

Supplementary Table S5: Evaluation of the mediating or confounding effect of gender on caffeine intake and suicidality outcomes

Author	Year	Country	Study population	Key findings†
Evren	2015	Istanbul	4957 10th grade students with a mean age of 15.56 years old (± 2.74 years old), across 45 schools from 15 districts in Istanbul, were evaluated through a cross-sectional online self-report survey.	Compared to those who do not consume energy drinks, those who consumed energy drinks once in their lifetime were more likely to be male (OR = 1.61, 95%CI = 1.41, 1.85).<br><br>Compared to those who do not consume energy drinks, those who consumed energy drinks once to three times in a month were more likely to be male (OR = 3.09, 95%CI = 2.66, 3.60).<br><br>Compared to those who do not consume energy drinks, those who consumed energy drinks once to five times in a week were more likely to be male (OR = 5.97, 95%CI = 4.20, 8.48).<br><br>Compared to those who do not consume energy drinks, those who consumed energy drinks everyday were more likely to be male (OR = 5.87, 95%CI = 3.47, 9.94).
Masengo	2020	Canada	5,538 students from grades 7 to 12, with a mean age of 15.3 years, were evaluated as part of the 2017 Ontario Student Drug Use and Health Survey (OSDUHS)	Compared to those with suicidal thoughts who do not consume energy drinks, those with suicidal thoughts who consumed energy drinks in the past 12 months were more likely to be male (aIRR = 3.34, 95%CI = 2.20, 5.08) than female (OR = 1.53, 95%CI = 1.10, 2.15).<br><br>Compared to those with suicide attempts who do not consume energy drinks, those with suicide attempts who consumed energy drinks in the past 12 months were more likely to be male (aIRR = 4.37, 95%CI = 2.27, 8.41) than female (OR = 3.27, 95%CI = 1.86, 5.76).

S. Park	2016	South Korea	68,084 adolescents aged 12-18, with a mean age of 15.09 years, were evaluated in a cross-sectional study in 2015, with data taken from the 2015 Korean Youth Risk Behaviour Web0-Based Survey.	Compared to those who had energy drinks less than once a week, those who had energy drinks 5 times or more per week were more likely to be male (OR = 1.79, 95%CI = 1.75, 1.82).
Kwak	2021	South Korea	267,907 middle and high school students with a mean age of 15.0 at the time of assessment were evaluated in a cross-sectional study, with data taken from the Korean Youth Risk Behaviour Web-Based Survey from 2014-2017.	Male participants were more likely to consume highly caffeinated beverages (HCB) (OR = 1.25, 95%CI = 1.20, 1.31).

Abbreviations: aOR, adjusted odds ratio; OR, odds ratio; CI, confidence interval; HR, hazard ratio

†Outcomes of interest include logistic or linear regression analysis for any association between gender on caffeine intake and suicidality outcomes

Supplementary Table S6: Quality assessment of included cohort studies using the Joanna Brigg's Institute Critical Appraisal tool

Study	1	2	3	4	5	6	7	8	9	10	11
Baethge	Y	Y	U	Y	Y	N	Y	Y	U	U	Y
Kawachi	Y	Y	Y	Y	Y	Y	Y	Y	Y	NA	Y
Lucas	Y	Y	Y	Y	Y	Y	Y	Y	Y	NA	Y
H. Park	Y	Y	Y	Y	Y	N	Y	NA	NA	NA	Y
Tanskanen	Y	Y	Y	Y	Y	Y	Y	Y	Y	NA	Y
Klatsky	Y	Y	Y	Y	Y	Y	Y	Y	Y	NA	Y
Cho	Y	Y	Y	Y	Y	N	Y	NA	NA	NA	Y
Evren	Y	Y	Y	Y	Y	N	Y	NA	NA	NA	Y
H. Kim	Y	Y	Y	Y	Y	N	Y	NA	NA	NA	Y
J. S. Kim	Y	Y	Y	Y	Y	N	Y	NA	NA	NA	Y
S. Y. Kim	Y	Y	Y	Y	Y	N	Y	NA	NA	NA	Y
Masengo	Y	Y	Y	Y	Y	N	Y	NA	NA	NA	Y
Mash	Y	Y	Y	Y	Y	N	Y	NA	NA	NA	Y
S. Park	Y	Y	Y	Y	Y	N	Y	NA	NA	NA	Y
Kwak	Y	Y	Y	Y	Y	N	Y	NA	NA	NA	Y
Akanni	Y	Y	Y	Y	Y	N	Y	NA	NA	NA	Y

Beauchamp	Y	Y	Y	Y	Y	Y	Y	NA	NA	NA	Y

Checklist
1. Were the two groups similar and recruited from the same population?
2. Were the exposures measured similarly to assign people to both exposed and unexposed groups?
3. Was the exposure measured in a valid and reliable way?
4. Were confounding factors identified?
5. Were strategies to deal with confounding factors stated?
6. Were the groups/participants free of the outcome at the start of the study (or at the moment of exposure)?
7. Were the outcomes measured in a valid and reliable way?
8. Was the follow up time reported and sufficient to be long enough for outcomes to occur?
9. Was follow up complete, and if not, were the reasons to loss to follow up described and explored?
10. Were strategies to address incomplete follow up utilized?
11. Was appropriate statistical analysis used?

Legend:
Y – Yes
N – No
U – Unclear
NA – Not applicable

Supplementary Table S7: Certainty of evidence assessed using the GRADE framework

Outcome	Exposure/Mechanism	Study Design	Risk of Bias	Inconsistency	Indirectness	Imprecision	Publication Bias	Grade	Notes
Suicide	Coffee	Mixed (Cohort + Cross-sectional)	Not serious (downgraded)	Serious (downgraded)	Not serious	Not serious	Undetected	Low	Evidence pooled from different study designs, increasing heterogeneity and risk of bias.
Suicide	Energy Drinks	Mixed (Cohort + Cross-sectional)	Not serious (downgraded)	Serious (downgraded)	Not serious	Not serious	Undetected	Low	Evidence pooled from different study designs, increasing heterogeneity and risk of bias.
Suicidal Ideation	Energy Drinks	Mixed (Cohort + Cross-sectional)	Not serious (downgraded)	Serious (downgraded; I^2>90% indicates substantial heterogeneity)	Not serious	Not serious	Undetected	Very low	High heterogeneity reduced confidence in the pooled estimate.
